# Alterations of retinal pigment epithelium cause AMD-like retinopathy in senescence-accelerated OXYS rats

**DOI:** 10.18632/aging.100243

**Published:** 2010-12-12

**Authors:** Anton M. Markovets, Valeriya B. Saprunova, Anna A. Zhdankina, Anzhella Zh. Fursova, Lora E. Bakeeva, Natalia G. Kolosova

**Affiliations:** ^1^ Institute of Cytology and Genetics SB RAS, Novosibirsk, Russia; ^2^ Belozersky Institute of Physico-Chemical Biology, Moscow State University, Moscow, Russia; ^3^ Institute of Mitoengineering, Moscow State University, Moscow, Russia; ^4^ Siberian State Medical University, Tomsk, Russia

**Keywords:** age-related macular degeneration, OXYS rats, molecular mechanism of pathogenesis, vascular endothelial growth factor, pigment epithelium-derived factor

## Abstract

Pathogenesis of age-related macular degeneration (AMD), the leading cause of blindness in the world, remains poorly understood. This makes it necessary to create animal models for studying AMD pathogenesis and to design new therapeutic approaches. Here we showed that retinopathy in OXYS rats is similar to human AMD according to clinical signs, morphology, and vascular endothelium growth factor (VEGF) and pigment epithelium-derived factor (PEDF) genes expression. Clinical signs of retinopathy OXYS rats manifest by the age 3 months against the background of significantly reduced expression level of VEGF and PEDF genes due to the decline of the amount of retinal pigment epithelium (RPE) cells and alteration of choroidal microcirculation. The disruption in OXYS rats' retina starts at the age of 20 days and appears as reduce the area of RPE cells but does not affect their ultrastructure. Ultrastructural pathological alterations of RPE as well as develop forms of retinopathy are observed in OXYS rats from age 12 months and manifested as excessive accumulation of lipofuscin in RPE regions adjacent to the rod cells, whirling extentions of the basement membrane into the cytoplasm. These data suggest that primary cellular degenerative alterations in the RPE cells secondarily lead to choriocapillaris atrophy and results in complete loss of photoreceptor cells in the OXYS rats' retina by the age of 24 months.

## INTRODUCTION

Age-related macular degeneration (AMD), the leading cause of blindness in the world, is under intensive investigation but its pathogenesis remains poorly understood. According to the clinical signs, it is usually defined two forms of this disease: atrophic (80-90%) and exudative (about 10% of cases). Atrophic (or dry) form is characterized by progressive degeneration of retinal pigment epithelium (RPE) and photoreceptor cells. Exudative (or wet) form is characterized by edema, spot bleeding, inflammation and choroidal neo-vascularization (CNV) which is a cause of ~90% of blindness from AMD. The RPE is a polarized monolayer of epithelial cells that separates the neural retina and the choroidal blood supply and forms a highly selective barrier fundamentally important for maintaining the health and integrity of the photo-receptors [1,2]. For several reasons, degeneration of RPE can create prerequisites for disease development and cause an alteration of choroid vessels [3,4]. RPE cells secrete many regulatory factors, including VEGF and PEDF [1,5-8]. It is well established that vascular endothelial growth factor A (VEGF-A), the member of a large family of growth factors, is a key regulator of physiological and pathological angiogenesis and vascular permeability [9,10].

Experimental and clinical studies showed that VEGF is increased in retinal pigment epithelium (RPE)/choroid complex of AMD patients [8,11]. An increase of VEGF gene expression launches angiogenesis and is a sufficient condition for CNV development [12,13]. New vessels cause edema, hemorrhages, and exudates due to the ability of VEGF to increase vascular permeability [8,9]. Application of anti-VEGF agents showed con-vincing results in studies aimed at delaying CNV development in human subjects [14]. Pigment epithelium-derived factor (PEDF), the most potent inhibitor of angiogenesis [15], serves as an antagonist of VEGF and the balance of these two factors regulates angiogenesis in retina [16,17]. It is known that a decreased level of PEDF in retina is associated with several proliferative diseases of the eye [13,18] and it may be an etiologic factor of AMD and be involved in switching from the dry to wet form. Additionally, it has been shown that VEGF and PEDF cross-regulate each other [19]. There are data about association of late stage of AMD with some genes, including CD36, which seems to play an important role in maintaining chorioretinal homeostasis [20]. But little is known about the involvement of VEGF and PEDF in formation of dry form of AMD due to difficulty of investigation this stage in respect to people.

Animal models are a useful tool for studying etiology and pathogenesis of the disease and to test new therapeutic approaches and estimate their efficacy. There is mounting evidence that senescence-accelerated OXYS rats may be a suitable model of AMD [21-23]. According to funduscopy, signs of retinopathy, which in their clinical manifestations similar to those observed in human AMD, develop in OXYS rats to the age of 4 -5 months. Exploration of retinal function by electroretinography had indicated reducing level of b-wave of scotopic electroretinogram in OXYS rats which progressed to complete loss of photoreceptors by 24 months [23]. Histological study showed that choroid vessels, pigmented epithelium, and radial glia are most vulnerable to injury in the OXYS rat's retina [22,24], but molecular mechanisms of retinopathy development in OXYS rats were not investigated as well as the dynamics of the clinical manifestations of disease. The aims of this study were to explore age-related characteristics of retinopathy in OXYS rats and to identify distinct stages in its development based on structural analysis of retina and expression of VEGF and PEDF.

## RESULTS

### Ophthalmoscopy examination

**Figure 1.**
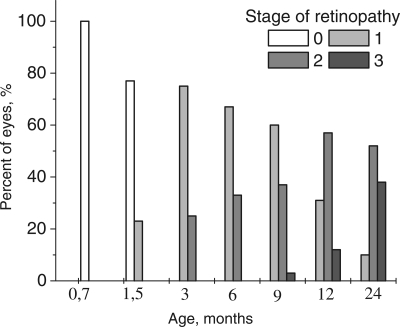
Distribution of the stages of development of retinopathy in eyes of OXYS rats. 0 − 3 – corresponding stage of the retinopathy according to Age-Related Eye Disease Study grade protocol (eyephoto.ophth.wisc.edu). 790 animals were examined in total.

Retinas from 20 day-aged OXYS rats did not revealed the presence of any pathological signs. The first AMD-like alterations in the fundus of the eye were found in 22% of the OXYS rats when they reached the age of 1.5 months (Figure 1). Alterations manifested itself as distorted reflectance of the fundus, swelling of the retina, the emergence of distinct foci of ischemia, and the signs of atrophy of choriocapillaris and RPE.

Up to the age of 3-4 months, the morbidity had already reached 100% when OXYS rats exhibited the prevalent 1st stage of the disease: drusen with signs of atrophy of RPE and partial loss of choriocapillaris, whereas the major blood vessels of choroid remained unchanged. In some animals, we observed detachment of pigment epithelium in the form of dome elevation in the posterior pole of the eye. Four to five percent of the animals at this age have features of the 2nd stage: large soft drusen, edema in the central zone, enlargement of the zone, exudative detachment of pigmented and neuroepithelium of the retina. The proportion of animals with this stage of the disease increased with age (Figure 1), and by the age of 12 months, it reached 31%.

Up to the age of 12 months, clinical symptoms of retinopathy worsened. The third stage was registered in 3% of animals at 9 months, but at 12 months, it was apparent in 44% of the animals and was characterized by severe irreversible changes. The distinctive signs of this stage are hemorrhagic detachment of retina, dotted bleeding in retina, and new blood vessel formation and cicatrization. Importantly, neovascularization did not develop in all animals, but rather, in isolated cases, as it normally happens in people. By the age of 24 months, the changes were irreversible in nearly all animals. In contrast, first alteration in Wistar retina started to develop by the age of 24 months and could not be strictly classified as signs of AMD.

### Histological analysis and electron microscopy

**Figure 2.**
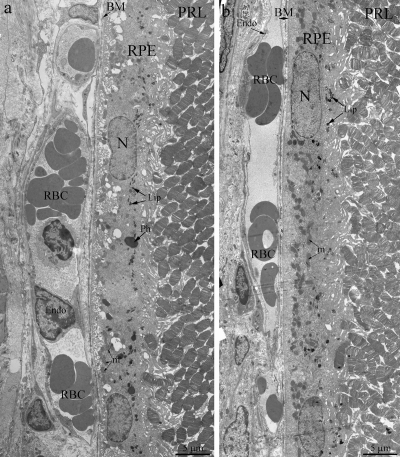
Outer retina of Wistar (**a**) and OXYS (**b**) rats at the age of 3 months. PRL – photoreceptors, RPE – retinal pigment epithelial cell, N – RPE nucleus, m – mitochondrion, Ph – phagosome, Lip – lipofuscin material, BM – Bruch's membrane, Endo – endothelial cell, RBC – red blood cell.

Light- and electron microscopy did not relive significant differences in structure of retina of OXYS and Wistar rats at 20 days and 3 months. The RPE layer has one line of cells with normal localized nucleus, endoplasmic reticulum, ribosome and mitochondria (Figure 2). On the Figure 2 endothelial cells, which formed the wall of choriocapillaries, cavity of choriocapillaries and erythrocyte in it are good visible. But already at the age of 20 days, along with normally functioning choriocapillaries, we observed appearance choriocapillaries with stasis of blood cells. (Quantity analysis of choroid vessels and RPE cells see below).

**Figure 3.**
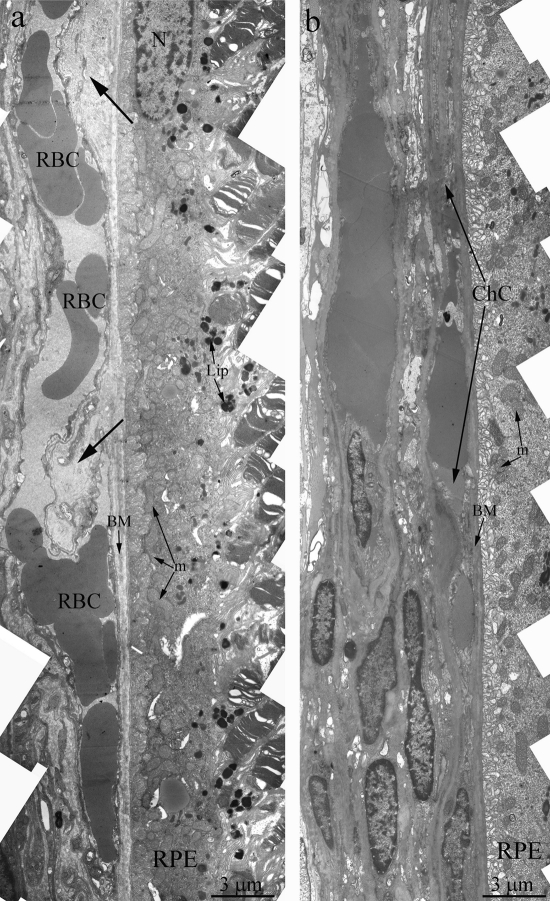
Outer retina of Wistar (**a**) and OXYS (**b**) rats at the age of 12 months. Arrows show thickening of Brunch membrane. RPE – retinal pigment epithelial cell, N – RPE nucleus, m – mitochondrion, BM – Bruch's membrane, ChC – choriocapillaris complex, RBC – red blood cell.

Towards the 12 months of age, destructive changes in OXYS rat's retina progressed and affect RPE/ Bruch's/choriocapillaris complex in contrast to Wistar rats (Figure 3a) with small anomaly in Bruch's membrane ultrastructure and open choriocapillaries. Main ultra-structural alterations are revealed in the cell apical part. First of all, this is the absence of a continuous layer of electron-dense inclusions as well as practically completes absence of phagosomes – debris of the photoreceptor outer segments phagocytized by pigment epithelium, whirling extensions of the basement membrane into the cytoplasm (Figure 3b). Underlying these areas was pronounced thickening of Bruch's membrane with disturbance of collagen and elastin layers. The choriocapillaris was found to have severe endothelial degeneration and transformation to fibrous tissue in the most severely affected regions.

**Figure 4.**
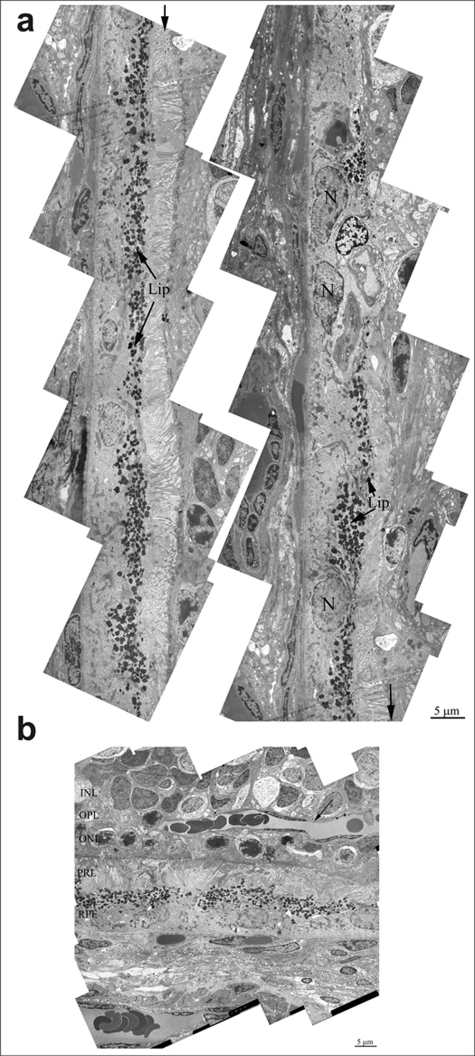
(**a**) Ultrastructure of pigment epithelium of 25-months-old OXYS rat. Selected area is shown under high magnification. Lip – lipofuscin material, N – retinal pigment epithelial cell nucleus. (**b**) Region of pigment epithelium of 25-months-old OXYS rat. Retina injure was estimated as 3 units. INL – inner nuclear layer, where the body and nucleus of interneurones is lied; OPL – outer plexiform layer – where axon of photoreceptor and dendrite of interneurone are in contact; ONL – outer nuclear layer, body and nucleus of photoreceptors; PRL – photoreceptors, RPE – retinal pigment epithelial cell. Arrow shown the blood vessel.

**Figure 5.**
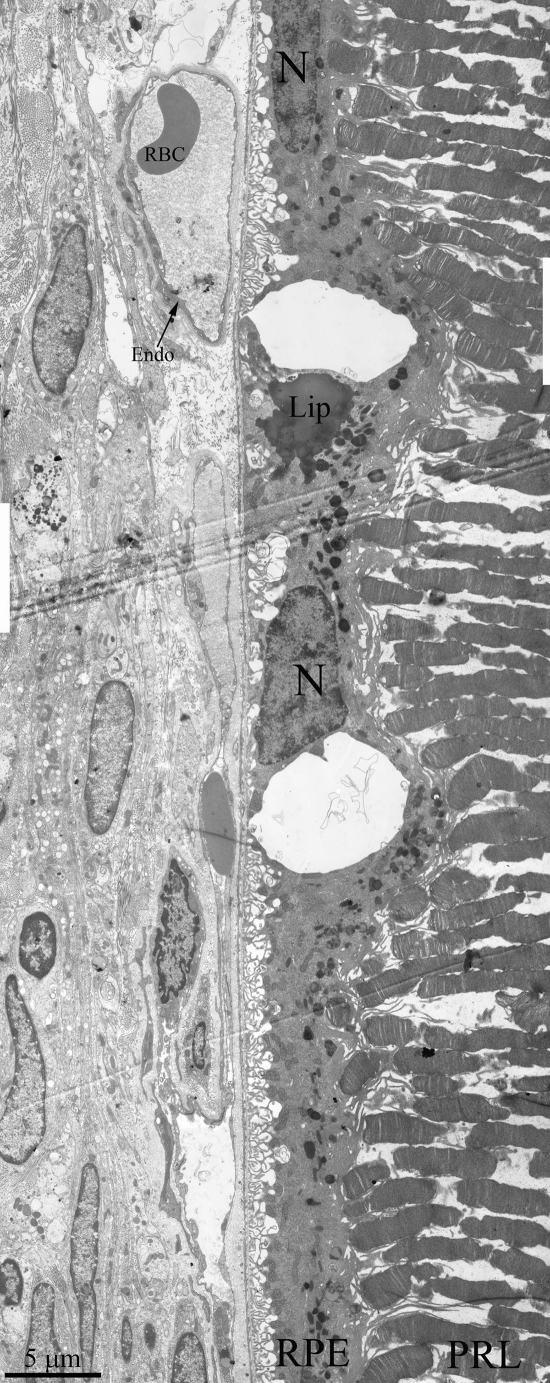
Ultrastructure of outer retina of Wistar rat at the age of 25 months. PRL – photoreceptors, RPE – retinal pigment epithelial cell, N – RPE nucleus, Lip – lipo-fuscin material, BM – Bruch's membrane, Endo – endothelial cell, RBC – red blood cell.

In the retina of 24 months-aged OXYS rats, there is almost complete fibrosis of choroid and destruction of endothelium and, as a result, obliteration of cavity of choriocapillaries. Ultrastructure analysis of OXYS eyes from 24-month-old animals shown, that area of degenerating retina is shown from the inner nuclear layer to retinal pigment epithelium/Bruch's/ choriocapillaris complex (Figure 4b). Outer nuclear layer and the photoreceptors are disorganized and fragmented. Changes of the RPE included significantly increased number of lipofuscin granules, atrophy of some RPE cells and vascularization (Figure 4a). Really, as the photoreceptor cells degenerated and the outer nuclear layer and photoreceptor cell layers disappeared, vessels migrated toward the RPE (arrow on Figure 4a). Thickening of Brunch membrane with disturbance of collagen and elastin layers were seen in all eyes (Figure 5a).Also there is almost complete fibrosis of choroid and destruction of endothelium and, as a result, obliteration of cavity of choriocapillaris.

Ultrastructure of Wistar retina had some changes that typical for aging, but it was not so dramatic pathology (Figure 5) as in OXYS rats. We can see all outer retina layers. It is also increased amount of lipofuscin granules. Cavity of most choriocapillaries decreased, but vessels are functionally active, as it visible.

**Figure 6.**
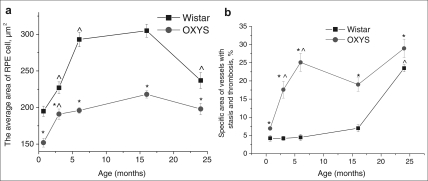
Age-related changes of the average area of RPE cells **(a)** and specific area of vessels with stasis and thrombosis **(b)** in the retinas' sections of OXYS and Wistar rats. *interstrain differences; ^differences in comparison with previous age; p<0.05. Data presented as mean ± S.E.M.

Quantitative analyses of the morphometry data showed that the average area of RPE cell was affected both by age (F_4.414_= 15.8, p<0.000) and by genotype (F_1.39_=73.4, p<0.000) and there was also a significant interaction between these two factors (F_4.414_=3.7, p<0.007). Overall, we observed well-pronounced growth with age the average area of RPE cells in both OXYS and Wistar rats from 20 days to 17 months of age (1.4- and 1.6-fold respectively, p<0.000). By the age of 24 months, the area extent of RPE cells decreased slightly compared to 17-month-old animals, but the decline was significant only in the Wistar rats (p<0.00004), while in the OXYS strain, we detected only a tendency for lowering (p<0.056) as shown on Figure 6a.

Pairwise comparisons within the strains revealed reduction of average area of RPE cell already in 20-day-old OXYS rats by 22% compared to Wistar rats (p<0.000). At ages 3, 6, 17 and 24 months, the differences remained and this parameter was lower in OXYS rats by 15% (p<0.023), 33% (p<0.000), 28% (p<0.001) and 16% (p<0.026), respectively.

**Figure 7.**
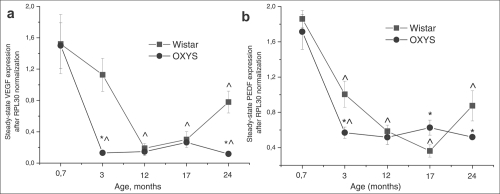
Messenger RNA (mRNA) expression of (**a**) vascular endothelium growth factor (VEGF) and (**b**) pigment epithelium-derived factor (PEDF) in retina determined by real-time polymerase chain reaction (PCR), N=5. Data presented as mean ± S.E.M. *interstrain differences; ^differences in comparison with previous age; p<0.05.

ANOVA analysis of morphometry data revealed age- related increasing of the specific area of choriocapillaris with stasis and thrombosis (F_4.68_= 22.4, p<0.000), which was observed in rats of both strains. In addition, its quantity was affected by genotype (F_1.68_=56.7, p<0.000), and since the age of 20 days, specific area of vessels with signs of partial occlusion of retinal vessels was greater in OXYS rats compared to the age-matched Wistar controls (Figure 7b). There was also a significant interaction between these two factors (F_4.68_=4.8, p<0.002). As shown in the Figure 6b, in the Wistar retina, the number of vessels with thrombosis and stasis remained unchanged between 20 days and 17 months of age. Only at age 24 months the percentage of choriocapillaris with signs of abnormality became 5.6-fold greater than that in the 20-day-old animals.

OXYS rats exhibited a relatively rapid expansion of specific area of choriocapillaris with stasis and thrombosis already by age 6 months. Up to the age of 24 months, these pathological changes progressed, but at a slower rate. As a result, at ages 3, 6, and 24 months, this parameter in the OXYS retina was 2.6-, 3.6-, and 4-fold higher compared to 20-day-old OXYS rats (p<0.006, p<0.0002, p<0.0001, respectively).

### Gene Expression

Expression of VEGF gene was affected both by age (F_4.39_= 18.3, p<0.000) and by genotype (F_1.39_=10.0, p<0.003) and there was also a significant interaction between these two factors (F_4.39_=3.3, p<0.020). The same was observed for expression of PEDF gene: age – F_4.39_=46.9; p<0.000; genotype – F_1.39_=4.5; p<0.040. Factors also interacted with each other (F_4.39_=3.1; p<0.026), which is probably due to the differences in age-dependent changes of retinal VEGF and PEDF expression between Wistar and OXYS strains. *Post hoc* analysis revealed that the maximum level of genes mRNA was in the retina at the age of 20 days (Figure 6) and had not difference between strains. Expression of VEGF and PEDF genes decreased 10-fold (p<0.000) and 5-fold (p<0.001) respectively between 20 days and 24 months of age in both Wistar and OXYS rats. At the same time, in OXYS rats, the reduction progressed more rapidly than in Wistar (Figure 7).

At the age of 20 days, there was no differencebetween Wistar and OXYS rats in terms of expression of VEGF and PEDF. But already at the age of 3 months, VEGF expression (Figure 7a) in OXYS rats was almost 10-fold lower (F_1.8_=21.9, p<0.002) compared to 20-day-old animals and to age-matched Wistar rats (F_1.8_=21.7, p<0.002) and PEDF expression (Figure 7b) decreased three-fold (p<0.0001) from age 20 days to 3 months and was about two-fold lower (F_1.8_=7.29; p<0.027) compared to the age-matched Wistar animals (F_1.8_=7.29; p<0.027). Expression of both genes in OXYS rats remained without significant changes throughout the rest of the observation period.

VEGF expression at the age of 24 months was 4- (p<0.002) and 2.6-fold higher (p<0.008) than in 12 and 17-month-old Wistar rats respectively, and it reached half of the expression level at the age of 20 days (p<0.0003). By the age of 24 months, PEDF expression increased by 2.4-fold (p<0.013) compared to 17-month-old Wistar rats (Figure 7).

## DISCUSSION

Based on the results of the ophthalmoscopic examination we can define two periods for development retinopathy in OXYS rats. The first period is manifestation of early disease signs from 1.5 to 3 months of age. The second period from 12 to 14 months corresponds to advanced stage of retinopathy. Our morphological investigation confirmed that the same retinal structure is involved in pathological processes in OXYS rats as in humans AMD [25,26]. Primary cellular degenerative alterations have been observed first in the RPE cells of OXYS rats, which secondarily lead to damage in the choriocapillaries and photoreceptor cells.

It is extremely important that we found changes in the OXYS retina already at the age of 20 days, when any obvious clinical signs are absent. Fist alteration of RPE cells in the retina of OXYS rats begin at the age of 20 days, are aggravated to 3-6 months by disturbances of microcirculation, and at this age correspond to 24-month-old Wistar rats. As RPE is pivotal for maintaining the structure and function of the retina, the early alteration of RPE cells may be a key initiating factor for retinopathy development in OXYS rats and a cause of all subsequent pathological changes. Clinical and experimental data suggest that normal aging processes can lead to structural and blood flow changes that can predispose an individual to AMD, although advanced age does not inevitably cause AMD [27,28]. Thus our investigation has shown that particularly age-related changes of retinal ultrastructure in Wistar and OXYS rats are non-specific, generally speaking, but the age of onset and rate of retinopathy development differ sharply between these strains. At the same time in support of our previous reports [24], the present results indicate evidence of impaired of phagocytic function of RPE in the adult 12 months-old OXYS rats. One of the main functions of the RPE is the phagocytosis of shed photoreceptor outer segments. Photoreceptors are exposed to intense levels of light, thus leading to accumulation of photo-damaged proteins and lipids [1]. As opposed to Wistar rats in OXYS rats lipofuscin granules are accumulated in the base of the RPE cell offshoots and fill in the space between rod cells, thus contributing to the development of further disturbances in the visual cycle functioning. Practically complete absence of phagosomes also points to disturbances in functioning of photoreceptor membranes in OXYS rats' retina. It is known that experimental destruction of RPE cause atrophy of choriocapillaries in rat's retina [4] and we observed the same phenomenon in the OXYS retina: with increasing age, the combined atrophy of RPE cells and choriocapillaris. The relationship between RPE and capillary endothelium can be mediated by VEGF signaling [10].

We can hypothesize that the accelerated decrease of VEGF and PEDF genes expression at the mRNA level in OXYS rats is due to early RPE dysfunction because both of these genes are primarily expressed in RPE cells. Also recently it was shown [29] that loss of polarity of RPE cells in AMD may results in alteration of PEDF, VEGF expression and lead to marked loss of neurotrophic and vascular support for the retina potentially leading to photoreceptor loss and blindness. This notion is supported by our data from morphological and ultrastructure analysis and examination of VEGF and PEDF genes expression. We found that expression of those genes decreased with age in both rat strains, with the maximum around 20 days of age. In this case, our data confirms the results of Steinle and coworkers [27] who observed a decrease of VEGF and PEDF gene expression during normal aging of retina.

We suppose that the age-related decrease of VEGF gene expression is premised for development signs of early AMD. Indeed, we found that the early stage of retinopathy in OXYS rats formed simultaneously with the significantly decreased expression level of VEGF and PEDF genes compared to control rats. That alteration of genes expression may disrupt physiologic feedback between RPE and choriocapillaries and lead to the subsequent atrophy of blood vessels, development of hypoxia in outer retina. In literature, there are many reports that support the existence of such a mechanism that provides fine-tuning of choriocapillaries for the necessary nutrient supply to the outer retina [8,10]. Most sensitive to hypoxia multifunctional RPE cells maintain complex structure of the whole retina [1] and its dysfunction is critical for development of retinopathy. There is a vicious circle: retinal ischemia results in damage to the pigment epithelium, which explains the reduced level of VEGF and PEDF gene expression, which in turn leads to the choriocapillaries atrophy, exacerbating ischemia, thereby contributing to the development of retinopathy.

We found an increase of VEGF and PEDF mRNA expression at age 24 months in Wistar rats, which is in good agreement with the results of Steinle [27]. VEGF gene expression gradually increased starting from 12 months of age, while the expression of PEDF from 17 months of age in these rats. Early signs of retinopathy became manifested in 24 month-old Wistar rats and such an upregulation of the expression of certain genes might be a compensatory reaction to the age-related changes in retina. It is known that VEGF and PEDF regulate expression of each other [19,30,31]. Thus, we can assume that by the age of 24 months, there were hypoxic conditions present in the Wistar retina due to age-related decrease of VEGF and it caused the upregulation of VEGF gene expression. On the other hand, VEGF promotes expression of PEDF gene and this may serve as a physiological regulatory mechanism that probably prevents switching from the dry to wet form and CNV. Those age-related changes may form the basis for AMD development.

It is interesting that at age 17 months, expression of VEGF and PEDF genes in the OXYS retina increased, same as in Wistar rats, but subsequently, the expression of those genes in OXYS rats declined to a rather low level. We propose that in OXYS rats, the compensatory mechanism that responds to retina damage is either suppressed or disrupted due to the impaired function of RPE cells. The decreased average area of RPE cells in OXYS rats in comparison with Wistar did not allow for an adequate compensatory response, and it led to almost complete degeneration of retina in OXYS rats by age 24 months. Nonetheless, the premise for this pathology was formed at a previous period of retinopathy in OXYS rats.

Advanced AMD is linked with increased VEGF gene expression, especially during development of CNV [11]. We did not find an increasing mRNA level of VEGF at age 12 months or older in OXYS rats (age of formation of developed retinopathy) compared to Wistar rats. Nevertheless, it increased in 17 month-old OXYS rats compared to its own baseline and, therefore, this observation is in good agreement with experimental data in humans. It might be supposed that the observed VEGF level is increased locally because the amount of RPE cells is decreased according to our histological investigation. To clarify this question, it would be necessary to perform an immunohistochemical study of the OXYS retina. Bhrutto and colleagues [18] have shown that the cause of the increased VEGF level is immune cells, which migrate to the retina.

The most important question now is what determines the switching between dry and wet form of AMD. DanHong Zhu et al. [32] found that bone morphogenetic protein-4 (BMP4) is differentially expressed in atrophic and neovascular AMD. Early they shown that BMP4 can induce RPE senescence *in vitro* [33], and that RPE chronically exposed to sublethal doses of oxidative stress can increase their BMP4 expression and exhibit a senescent phenotype, thus supporting the contention that BMP4 mediates oxidative stress-induced RPE senescence. The same situation may be in OXYS rats due to mitochondria dysfunction [23].

The causes of early alteration of RPE cells in OXYS rats are not yet clear. In the eye, the retinal pigment epithelium (RPE) is exposed to a highly oxidative environment, partly due to elevated oxygen partial pressure from the choriocapillaris and to digestion of polyunsaturated fatty acids from photoreceptor outer segments [34]. There is strong evidence for mitochondrial dysfunction being involved in AMD [35,36] and oxidative stress is the main injury mechanism. Thus, it is logical to suppose that the early decline of RPE in the retina of OXYS rats may be associated with genetic predisposition to mitochondrial dysfunction. This hypothesis is supported by one study which demonstrated significant destruction of mitochondria in RPE of OXYS rats [23]. Nonetheless, we had shown previously that there were no signs of energy deficiency in the brain of 3-months-old OXYS rats. Additionally, already at age 20 days, there are signs of adaptation to hypoxia in the brain without energy deficiency, for example, an increased amount of phosphocreatine in OXYS rats as compared to Wistar rats [37]. Retina develops as a part of the brain and it seems logical that mitochondrial dysfunction must be a consequence of some pathology in early ontogenesis. Be that as it may, clarification of this question would necessitate further studies of mitochondrial function in retinal tissue.

In the present and in a previous report [24] we observed redistribution of lipofuscin granules in RPE cells in 11- to 13-month-old OXYS rats. These granules are known to have a strong phototoxic potential mediated by light-dependent ROS generation and they could be a reason for further RPE degeneration. The observed pattern of redistribution of lipofuscin granules confirmed that the cellular functions of RPE in OXYS rats are either impaired or completely disrupted.

The set of clinical, morphological and molecular features of retinal degeneration in OXYS rats suggests that these animals are adequate model of AMD. This animal model could be used for studying pathogenesis of early stages of the disease including preclinical changes and to test therapeutic action of drugs. In this view, our previous experiments with OXYS rats supported therapeutic benefits of bilberry (Vaccinium myrtillus) extract [38]. Investigation carried out on OXYS rats, at first time had demonstrated unique therapeutic potential of the novel mitochondria-targeted antioxidant SkQ1, (10-(6'-plastoquinonyl) decyltriphenylphosphonium) for AMD treatment. We showed recently that addition of SkQ1 to the food or in the form of droplets completely is able to not only prevent retinopathy but also to reduce the severity of already developed pathological changes of retina in OXYS rats [23].

## METHODS

### Animals

The OXYS rat strain was developed at the Institute of Cytology and Genetics from Wistar stock by selection on the basis of susceptibility to cataractogenic effects of a galactose-rich diet and by inbreeding of highly susceptible rats as described earlier [39,40] and registered in the Rat Genome Database [41]. Today we have the 94nd generation of OXYS rats with spontaneously developing cataract and accelerated-senescence syndrome inherited in a linked manner. Cataract still serves as the key parameter for maintaining the phenotype of OXYS strain whereas the other signs of accelerated senescence, including retinopathy, appeared as concomitant.

Male OXYS and male Wistar (as controls) rats were born and reared at the Center for Genetic Resources of Laboratory Animals of Institute of Cytology and Genetics SB RAS (Novosibirsk, Russia). At the age of 4 weeks, the pups were taken away from their mothers and housed in groups of five animals per cage (57×36×20 cm) and kept under standard laboratory conditions (at 22±2^o^C, 60% relative humidity, and natural light), provided with a standard rodent feed, PK-120-1, Ltd. (Laboratorsnab, Russia), and given water ad libitum.

### Ophthalmoscopic examination

Ophthalmoscopic examination of OXYS and Wistar rats was carried out using a Beta direct ophthalmoscope (Germany) equipped with a slit lamp, after dilatation with 1% tropicamide. Assessment of stages of retinopathy was carried out according to Age-Related Eye Disease Study (AREDS) grade protocol (eyephoto.ophth.wisc.edu). In total, we examined 790 animals between 20 days and 24 months of age.

### Histological analysis and electron microscopy

A total of 42 OXYS and 37 age-matched Wistar rats were used for histological and morphometry studies. Animals were examined at the age of 20 days, 3, 6, 12 and 24 months (6 – 10 per group). For studies of the main parameters, we selected retinas on the basis of fundoscopy findings in OXYS rats. Morphological analysis was performed as described earlier [21]. The posterior wall of the eye was collected, fixed in 2.5% glutaraldehyde, postfixed in 2% OsO_4_, dehydrated in ascending concentrations of alcohol and in acetone, and embedded in epon-812. Semi-thin sections were sliced on an LKB-4 ultratome and stained with 0.1% toluidine blue. Counting was carried out on photo-graphs covering section regions from 50 to 250 mkm. RPE cells' area and specific area of choroid vessels were measured using quantitative analyses of the images were performed using Axiovision software (Zeiss, Thornwood, NY). Estimation was performed by examination 50 fields of view for each animal.

Ultrastructural study was performed on OXYS and Wistar rats at the age of 3, 12 and 24 months. Per 5 animals were in each group and in each of eye 3 areas were analyzed. Material for electron microscopy was fixed for 2 h at 4°C in 3% glutaraldehyde solution in buffer, pH 7.4. Then the material was additionally fixed with 1 % osmium tetroxide solution for 1.5 h and dehydrated in solutions of increasing alcohol concentrations (70% ethanol saturated with uranyl acetate). The material was embedded in Epon-812 epoxide resin. Serial ultrathin sections were prepared on a Leica Ultracut ultramicrotome (Leica, Austria) and stained with lead according to Reynolds [42]. The preparations were examined and photographed in a HU-1lB electron microscope (Hitachi, Japan).

### RNA extraction and real-time PCR

Analysis of steady-state mRNA expression of VEGF in the retina of OXYS rats was completed at 20 days, 3, 12, 17, and 24 months of age (Wistar strain as control, 5 animals in each age group). At the appropriate age, rats were decapitated and eyes were removed. Retina for gene expression analysis was separated from the other tissue, placed into tubes for RNA isolation and frozen in liquid nitrogen. All specimens were stored at -70°C prior to the analysis.

Total cell RNA was isolated from rat retina by TRI-Reagent (Ambion) according to the manufacturer's instructions. The amount of isolated RNA was evaluated by means of electrophoresis of 1 μl of each RNA sample in 1.5% agarose gel. RNA content was determined in each sample using spectrophotometry at 260 nm and also by absorbance ratios 260/280 nm and 260/320 nm. RNA was stored at -70°C. Contaminating genomic DNA was removed by treatment with DNase I (Promega, USA) according to the manufacturer's instructions and then by repeated RNA extraction with the phenol-chloroform mixture and pure chloroform followed by precipitation with propanol. Reverse transcription was performed using M-MLV Reverse Transcriptase (Promega, USA) following the manufacturer's protocol. For subsequent PCR, we used of 0.25–1.0 μl of the resulting cDNA mixture.

Aliquots (4 μl) from all cDNA samples were mixed and the “average” solution was used for preparation of calibration curves, which were used for determination of relative cDNA level for genes of interest and a reference gene in experimental samples.

Age-related changes of *vegf* and *pedf* gene expression were investigated using iCycler iQ4 real-time PCR detection system (Bio-Rad Laboratories, USA) and SYBR Green I dye (Molecular Probes, USA). The housekeeping gene *Rpl30* (encoding large ribosomal subunit protein 30) was used as a reference gene. The following primers were used: Rpl305′_ATGGTGGCTGCAAAGAAGAC_3′ and 5′_CAAAGCTGGACAGTTGTTGG_3′; vegf 5′_CTGGCTTTACTGCTGTACCTCCACC_3′ and 5′_GGCACACAGGACGGCTTGAA_3′; pedf 5′_GATTGCCCAGCTGCCTTTGACA_3′ and 5′_GGGACAGTCAGCACAGCTTGGATAG_3′.

The reaction mixture (final volume of 25 μl) contained the standard PCR buffer (67 mM Tris-HCl, pH 8.9, 16 mM (NH_4_)_2_SO_4_, 0.01% Tween 20, and 10 mM β-mercaptoethanol), MgCl_2_ (3 mM for *vegf*, 1.5 mM for RPL30, 3 mM for *pedf*), 0.2 mM dNTPs, SYBRGreen I (1: 20,000 dilution), 150 nM primers, and 0.8 U of Taq polymerase (Institute of Cytology and Genetics, Russia). Reaction was carried out under the following conditions: heating at 95°C for 3 min, 40 cycles: denaturation at 95°C for 20 sec, annealing 20 sec, elongation at 72°C 30 sec; data collection was based on fluorescence for Rpl30 at 84°C for 30 sec, data collection by fluorescence for *vegf* and *pedf* genes at 87°C for 10 sec. After completion of PCR, the melting curves for specificity control were recorded. In each experiment, samples of investigated cDNA were mixed with primers for a gene of interest (four repeats per cDNA sample) in one microtube plate; similar samples were mixed with primers for the reference gene (also four repeats). “Standard” cDNA was diluted from 1:2 to 1:64 with the same primers (2-3 repeats). For each cDNA sample, PCR was repeated at least twice. To confirm amplicon size and reaction specificity, PAGE electrophoresis was performed with DNA molecular weight markers.

The initial level of expression of investigated cDNA was determined by standard calibration curves (versus “standard” cDNA) and this value was obtained for each gene of interest and normalized according to the amount of the reference gene cDNA [43].

### Statistical analysis

The data were analyzed using repeated measures ANOVA and nonparametric tests with the statistical package Statistica 6.0. Two-way ANOVA was used to evaluate the differences between OXYS and Wistar rats across ages (age × genotype). A Newman-Keuls *post hoc* test was applied to significant main effects and interactions in order to estimate the differences between particular sets of means. One-way ANOVA was used for individual group comparisons. Data are represented as mean ± S.E.M. Results were considered statistically significant if *p* value was less than 0.05.
